# Psychological reactance to vaccine mandates on Twitter: a study of sentiments in the United States

**DOI:** 10.1057/s41271-025-00554-0

**Published:** 2025-01-19

**Authors:** Pei-Hsun Hsieh

**Affiliations:** Center for Social Norms and Behavioral Dynamics, University of Pennsylvania, Philadelphia, PA 19104-6304 USA

**Keywords:** Vaccine mandates, Public health officials, Twitter, Sentiment analysis, Natural language processing

## Abstract

This study examines the relationship between vaccine mandates and public sentiment toward vaccines and health officials on Twitter. I analyzed 6.6 million vaccine-related tweets from July 2021 to February 2022 in the United States. Leveraging a large language model, BERT, I identified tweets discussing vaccine mandates even when lacking explicit keywords. Compared to non-mandate tweets, those mentioning mandates exhibit greater negativity, anger, and freedom-related language. Furthermore, increased state-level discussion of mandates correlates with rising levels of negativity and anger toward both vaccines and public health officials. Finally, greater disparity in vaccination progress across counties within a state is associated with increased anger in tweets directed toward both.

**Supplementary Information:**

The online version contains supplementary material available at 10.1057/s41271-025-00554-0.

## Key Messages

- Increases in negative sentiment and anger in tweets about vaccines and public health officials were linked to the prominence of vaccine mandate discussions.
- Mandatory vaccination policies should be used with caution, as they may generate public resistance and potentially weaken societal resilience in future crises.

**Supplementary Information:**

The online version contains supplementary material available at 10.1057/s41271-025-00554-0.

## Introduction

To increase vaccination coverage in the United States (US), various levels of government and private organizations have imposed vaccination requirements on targeted populations, spaces, and activities. Several US states introduced vaccination verification programs, such as vaccine passports, which were used to mandate vaccination for activities like interstate travel or indoor events [1]. Some states implemented vaccination requirements in specific occupations, such as healthcare workers. In addition to government mandates, many private businesses and organizations also implemented vaccine requirements for their employees and customers [2]. However, the mandates for the COVID-19 vaccines were met with resistance [3] and heated debates surrounding COVID-19 vaccines created a hostile environment for public health officials [4, 5]. Given the potential for compulsory immunization to intensify vaccine hesitancy and fuel anti-vaccine sentiment, the scientific community has urged caution in implementing vaccine mandates [6]. Brehm’s Psychology Reactance Theory [7] provides a theoretical framework for understanding why this increased anger toward vaccines and public health officials may occur. Psychology Reactance Theory posits that when individuals perceive an individual or group intending to control their behavior, a motivational state, psychological reactance, arises and compels individuals to regain a sense of freedom. People experiencing psychological reactance may feel angry as well as have negative thoughts [8], leading them to seek ways to reclaim their perceived freedom, both directly and indirectly.

Several studies have found evidence that mandating vaccination can trigger psychological reactance and reduce willingness to be vaccinated in some subpopulations. Sprengholz et al. [9, 10] found a positive correlation between psychological reactance from mandates and intention to avoid COVID-19 vaccines. Furthermore, Graeber et al. [11] and Schmelz and Bowles [12], using within-subjects designs, found that mandatory vaccination policies decreased self-reported willingness to vaccinate among a portion of German respondents compared to voluntary vaccination. Psychological reactance triggered by vaccine mandates may also lead to downstream behaviors such as anti-vaccine activism. Sprengholz et al. [10] found that individuals experiencing higher levels of reactance toward mandates reported increased intentions to engage in anti-vaccine activities, such as signing petitions and participating in demonstrations. This aligns with broader psychological research suggesting that individuals experiencing reactance may attempt to regain a sense of control by engaging in oppositional behaviors [13]. The aggressive response to mandates may extend to public health officials. Research by Topazian et al. [4] found a concerning rise in the justification of harassment and threats toward these officials during the pandemic. Public health officials were also targeted by conspiracy theories questioning their motives. For instance, Romer and Jamieson [14] found that nearly 20% of Americans believed the CDC inflated the threat of the virus to undermine President Trump. Dr. Anthony Fauci, then-director of the National Institute of Allergy and Infectious Diseases, became a particular focus of these conspiracy theories [15]. Twitter has played a significant role as an information channel during the pandemic [16]. Prior research has utilized Twitter to study vaccine hesitancy through sentiment analysis [17, 18]. For example, Bonnevie et al. [19] found that opposition to vaccines increased by 80% during the early stages of the pandemic.

The current study seeks to examine whether online sentiment toward vaccines and public health officials is associated with the salience of vaccine mandates, as suggested by Psychological Reactance Theory. This study has two main objectives. First, it aims to contribute to the literature on the consequences of mandatory vaccine policies by replicating findings related to psychological reactance using Twitter data, and to assess whether previous findings can be generalized across different data sources. The study specifically collected and analyzed vaccine-related tweets from the later stages of the pandemic, when state governments in the U.S. implemented mandatory vaccination policies. Second, the study explores the relationship between vaccine mandates and aggression directed toward public health officials. While some studies have documented increasing hostility toward public health authorities [4, 5], few have specifically investigated how mandatory vaccination policies may contribute to this aggression. There are three hypotheses in the study. First, perceived restriction of freedom is a necessary precondition for psychological reactance to occur, I hypothesize that the frequency of freedom-related words co-occurs with discussions of vaccine requirements. Second, given that individuals experiencing psychological reactance exhibit negative emotions and anger, I hypothesize that the level of negativity and anger expressed in tweets regarding vaccines correlates with discussions of vaccine requirements. Third, I hypothesize that these elevated levels of negativity and anger spill over into tweets directed at public health officials.

## Data and methods

This study employed the Bidirectional Encoder Representations from Transformers (BERT) model to automate tweet classification [20] to classify tweets about vaccine mandates from a larger database of all vaccine-related tweets. Supervised learning methods were employed for text classification to identify tweets related to vaccine mandates [21]. Unlike dictionary-based approaches, supervised learning offers the advantage of capturing implicit discussions about vaccine mandates. This is particularly valuable for tweets that do not contain explicit terms like “mandate” or “requirement.” For instance, a tweet like “So nobody upset that you can’t travel internationally without vaccinating?” would likely be missed by a dictionary approach, yet a supervised learning model can classify it as relevant due to the underlying context.

Analyses were conducted at two levels: the individual tweet level and the state-date level. At the tweet level, I compared tweets discussing vaccine mandates with vaccine-related tweets unrelated to vaccine mandates. At the state level, I used the proportion of mandate-related tweets among all vaccine-related tweets as a proxy for the salience of vaccine requirements within a state on a specific date. This proportion was then investigated for its association with aggregate levels of negativity and anger.

### Tweets about vaccines and public health officials

Tweets from July 2021 to February 2022 were collected using the Twitter Academic Research product track’s full-archive search endpoint (Twitter API v2). This endpoint retrieves historical public tweets that have met a search query since March 2006. The keywords to collect vaccine-related tweets include *vaccine*, *vaccines*, *vaccinate*, *vaccinates*, *vaccinated*, *vaccinating*, *vaccination*, *vaccinations*, *vaccinemandate*, and *vaccinemandates*; the keywords to collect tweets related to public health officials are *fauci*, *anthonyfauci*, *drfauci*, *#fauci*, *#anthonyfauci*, *#drfauci*, *#FireFauci*, *#ArrestFauci*, *cdc*, *#cdc*, *CDCgov*, and *Centers for Disease Control*. Tweets were then filtered to include only those in English and from the U.S., utilizing both tweet geotags and self-reported user locations [22]. This process resulted in a dataset of 6,655,234 tweets containing vaccine keywords, of which 5,836,423 could be geolocated to the state level. Similarly, 949,691 tweets containing public health authority keywords were identified and geolocated to the state level.

### Classifying mandate-related tweets

The BERTweet model [23], a BERT model pre-trained on tweets, was used to classify mandate-related tweets. To train machine learning models, a random sample of 3324 tweets was manually labeled by myself and research assistants. Each tweet was coded by two human coders, and in cases where the coders assigned different labels, a third coder provided the final label. Tweets discussing vaccination policies requiring inoculation to avoid restrictions (government- or private entity-imposed mandates for work or activities) were classified as mandate-related (see Supplementary Material Appendix A).

**Fig. 1:**
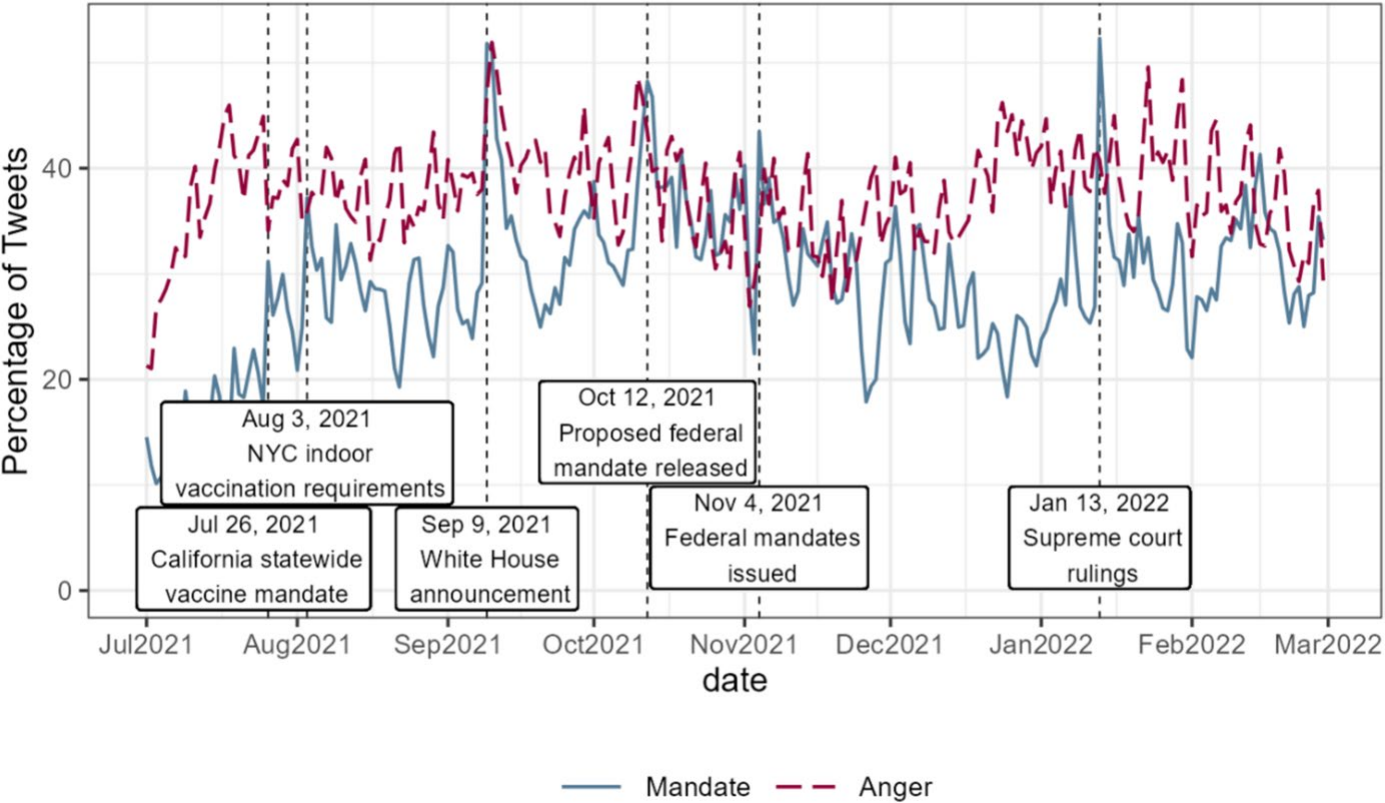
The daily percentage of mandate-related tweets and angry tweets, as classified by the machine learning models, among all vaccine-related tweets in the United States. The solid line and the dashed line represent the percentages of mandate-related tweets and angry tweets, respectively

BERTweet identified a total of 1,988,078 (29.87%) mandate-related tweets from all vaccine-related tweets. Figure 1 shows the percentage of mandate-related tweets out of all vaccine-related tweets over time. The trend reflects the major events related to vaccine mandates, including the first state-wide vaccine mandate in the U.S. issued by California, New York City’s indoor vaccine requirements, and the important dates of the federal mandates for healthcare workers and large employers. The global peak is January 13, 2022, when the Supreme Court ruled on the federal mandates.

### Negative sentiment in texts, anger in texts, and freedom-related words in texts

Negative sentiment within tweets was measured using the Valence Aware Dictionary and sEntiment Reasoner (VADER) [24] and TweetNLP’s sentiment analysis model [25]. VADER is a rule-based sentiment analysis lexicon specifically tuned for social media. VADER assigns negative sentiment scores not only based on words but also considers punctuation, emoticons, emoji, and capitalization. VADER calculates three scores from a text—negative, positive, and neutral—on a scale from 0 to 1. The scores were rescaled to 0 to 100 in order to match the range of other measures in the study. VADER also calculates the compound score calculated from the negative, positive, and neutral scores (on a scale from − 1 to + 1). TweetNLP is a pre-trained large language model built on an optimized version of BERT [26] and trained on a substantial dataset of tweets. It provides a range of fine-tuned models for various downstream tasks, including sentiment analysis. Its sentiment analysis model assigns one of three labels—positive, neutral, or negative—based on predicted probabilities.

Anger levels were measured by two methods: the percentage of words in the anger category from the Word-Emotion Association Lexicon [27] and TweetNLP’s multilabel emotion analysis model. The multilabel model allows a text to be labeled with multiple emotions for a single tweet. The machine-learning-based anger variable was measured by whether TweetNLP’s emotion analysis model labeled a tweet as an angry tweet. Tweets were coded for the presence of specific freedom-related words: *freedom*, *liberty*, *rights*, and *choice*.

### Statistical analysis

Given the large sample size of the data, the central limit theorem was applied, enabling hypothesis testing under the assumption that the probability distributions of the estimators approximate normal distributions. First, proportional tests were conducted to examine differences in proportions between mandate-related and non-mandate-related tweets for the following binary measures: (1) whether a tweet contained freedom-related words, (2) whether a tweet was classified as negative by a machine-learning-based method, and (3) whether a tweet was classified as angry by a machine-learning-based method. The alternative hypotheses were that mandate-related tweets were more likely to contain freedom-related words, be negative, and express anger compared to non-mandate-related tweets. Second, differences in means were tested for (1) the negativity score measured by a rule-based method and (2) the percentage of angry words identified by the rule-based method. The alternative hypotheses were that mandate-related tweets were more negative and angrier than non-mandate-related tweets. Additionally, Wilcoxon signed-rank tests were conducted as a robustness check for continuous measures measured by the rule-based methods.

### State-date panel data analysis

These analyses explored the relationship between the salience of vaccine mandates and the aggregate sentiment and emotion expressed in tweets concerning vaccines and public health officials. Tweets were aggregated by state and by date to identify periods, where vaccine mandates were highly salient by the percentage of mandate-related tweets within the total volume of vaccine-related tweets. The use of tweet volume as an indicator of issue salience is supported by prior research [28, 29].

The panel data (repeated measures) were analyzed with the emphasis on two dimensions: states (all 50 states and the District of Columbia) and days (243 days), totaling 12,393 observations. Given the structure of the data, I employed a two-way fixed effects model: state fixed effects to account for time-invariant state-level confounders (historical vaccine hesitancy, political orientation), and date fixed effects to control for time-specific confounders. Additionally, I used Driscoll and Kraay’s panel Newey–West type of standard errors [30], a semiparametric method, to address both cross-sectional and serial correlation in the models.

Nine regression models were estimated to investigate the relationship between vaccine mandate salience and various sentiments and emotions expressed in tweets. The key independent variable in all models is the percentage of mandate-related tweets out of all vaccine-related tweets in a state and date, serving as a proxy for the salience of vaccine requirements. The dependent variables are (1) the percentage of vaccine-related tweets containing a freedom-related word, (2) the mean negative sentiment score in vaccine-related tweets, (3) the mean percentage of anger words in vaccine-related tweets, (4) the mean negative sentiment score in tweets about public health officials, and (5) the mean percentage of anger words in tweets about public health officials. All models controlled for the following covariates: (1) the natural log of the number of vaccine-related tweets per person, (2) the natural log of the seven-day moving average of new COVID-19 cases and deaths per person to account for disease severity, (3) the natural log of the seven-day moving average of new COVID-19 vaccine doses per person and the percentage of the fully vaccinated population in a state to control for vaccination progress, and (4) the weighted standard deviation of the percentage of the fully vaccinated population across counties within a state to capture within-state polarization.

## Results

### Comparison between mandate-related and non-mandate-related tweets

**Fig. 2:**
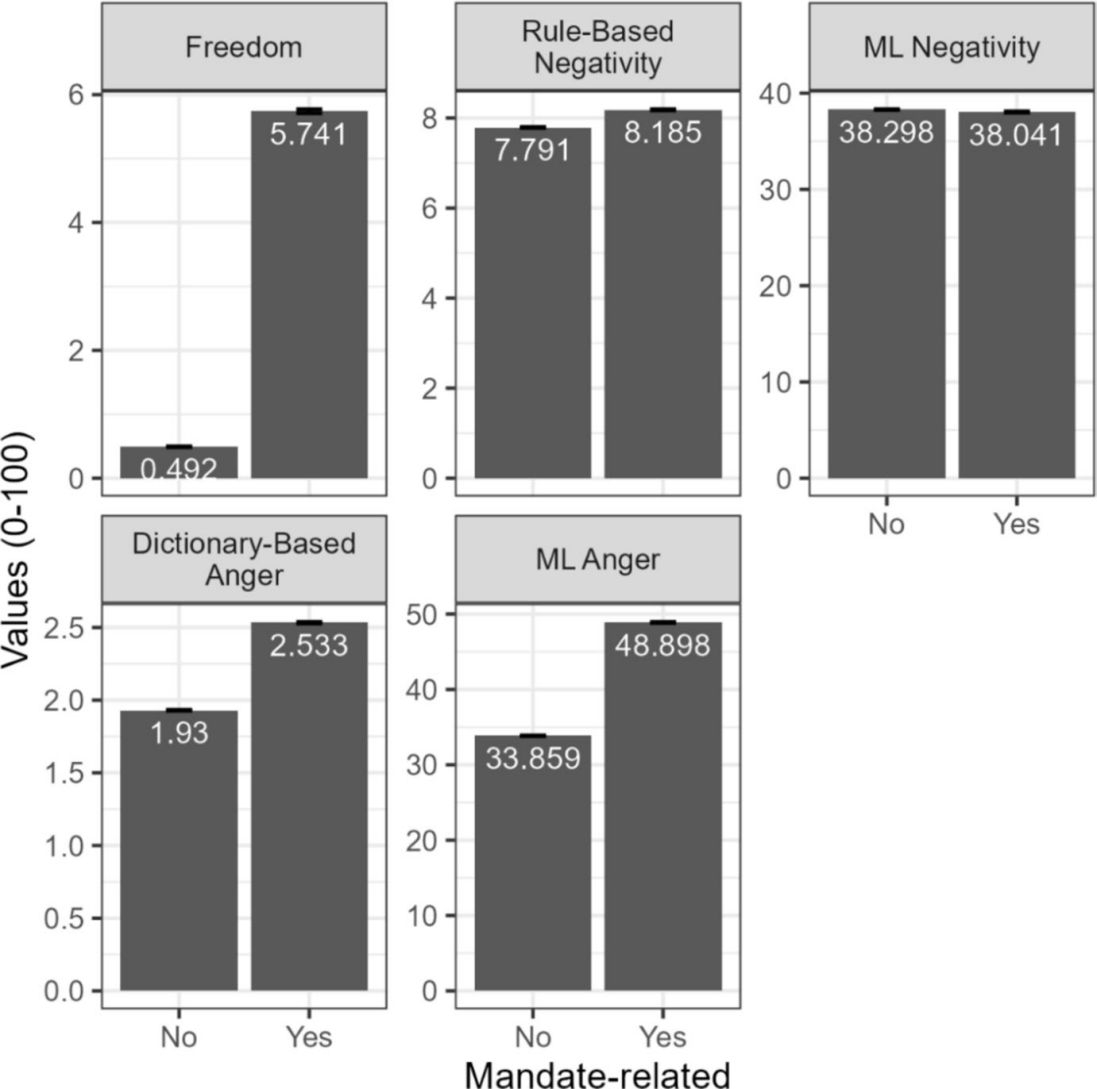
The comparison between mandate-related and other vaccine-related tweets. (Left): The percentage of tweets containing the following words about freedom: freedom, liberty, rights, choice (Middle): The mean of the VADER negative scores (from 0 to 100). (Right): The mean percentage of words in the NRCLex anger category (95% confidence interval for the error bars)

First, I compared mandate-related tweets to non-mandate-related tweets (see Fig. 2) because mandatory vaccination policies are more likely to induce psychological reactance than voluntary vaccination policies [9, 10]. The tweets about vaccine mandates were more likely to mention freedom, with 5.74% of mandate-related tweets containing one of the freedom-related words, compared to 0.49% of non-mandate tweets (one-tailed z-test for proportions, applied to a large sample size under the central limit theorem: Z = 312.24, *p* < 0.001). Tweets about vaccine mandates were also more negative when measured using a rule-based method (mandate-related: 8.2, non-mandate-related: 7.8, one-tailed z-test, applied to a large sample size under the central limit theorem: Z = 46.27, *p* < 0.001, one-tailed Wilcoxon signed-rank W = 4.8175^12^, *p* < 0.001). However, when negativity was measured using a machine-learning-based method, the percentages of negative tweets were nearly the same between mandate-related and non-mandate-related tweets (mandate-related: 38.04%, non-mandate-related: 38.298%, one-tailed z-test for proportions, applied to a large sample size under the central limit theorem: Z =  − 6.25, *p* ≈ 1).

Furthermore, anger levels, as measured by both the dictionary-based method and the machine-learning-based method, were higher among mandate-related tweets compared to non-mandate-related tweets. The average percentage of angry words was 2.53% for mandate-related tweets and 1.93% for non-mandate-related tweets (one-tailed z-test for a large sample size under the central limit theorem: Z = 184.67, *p* < 0.001; Wilcoxon signed-rank test: W = 5.029^12^, *p* < 0.001). Similarly, 48.898% of mandate-related tweets were labeled as angry by the machine-learning-based model, compared to 33.86% of non-mandate-related tweets (one-tailed z-test for proportions, applied to a large sample size under the central limit theorem: Z = 360.895, *p* < 0.001), consistent with the psychological reactance theory.

In addition, mandate-related tweets were, on average, less positive and more neutral than non-mandate-related tweets in terms of sentiments (Figure S1). I also found that the mandate-related tweets were more popular than other vaccine-related tweets. The mandate-related tweets had more likes, replies, and retweets than other vaccine-related tweets on average (Figure S2).

### State-date panel data analysis

**Fig. 3:**
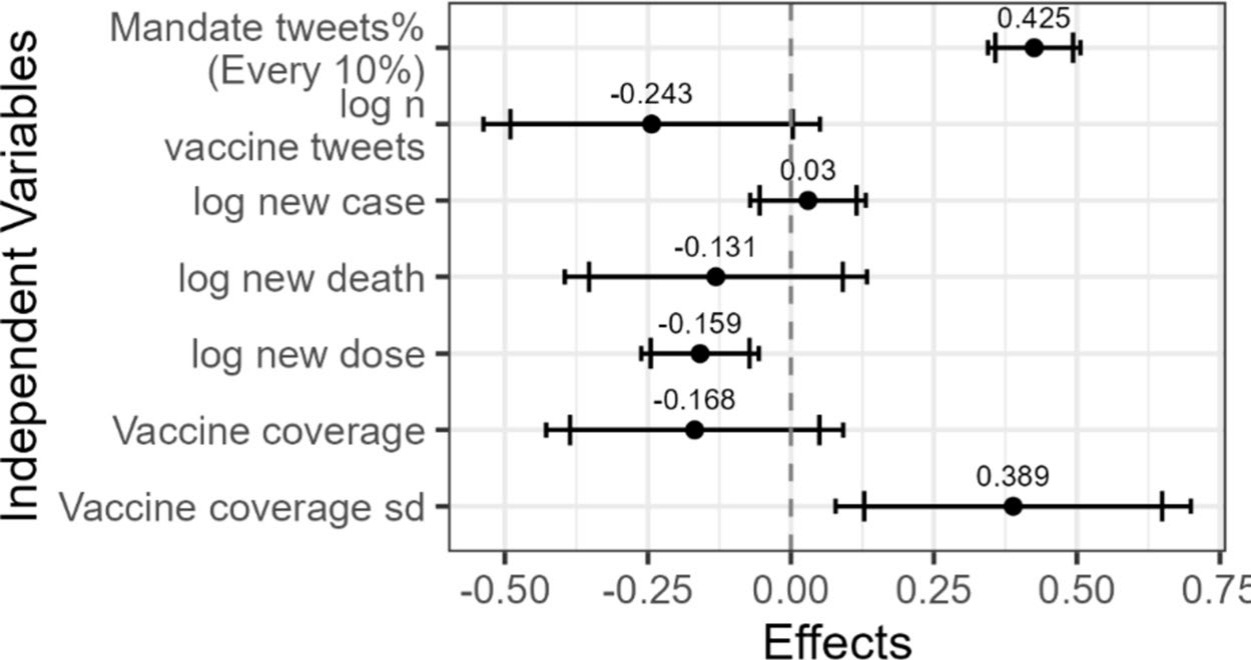
Regression results for the percentage of vaccine-related tweets containing freedom-related words. The dependent variable is the percentage of vaccine-related tweets that include a freedom-related word. The model controls for state and date fixed effects. Driscoll and Kraay’s panel Newey–West standard errors were applied. The wider and narrower error bars represent the 90% and 95% confidence intervals, respectively

**Fig. 4:**
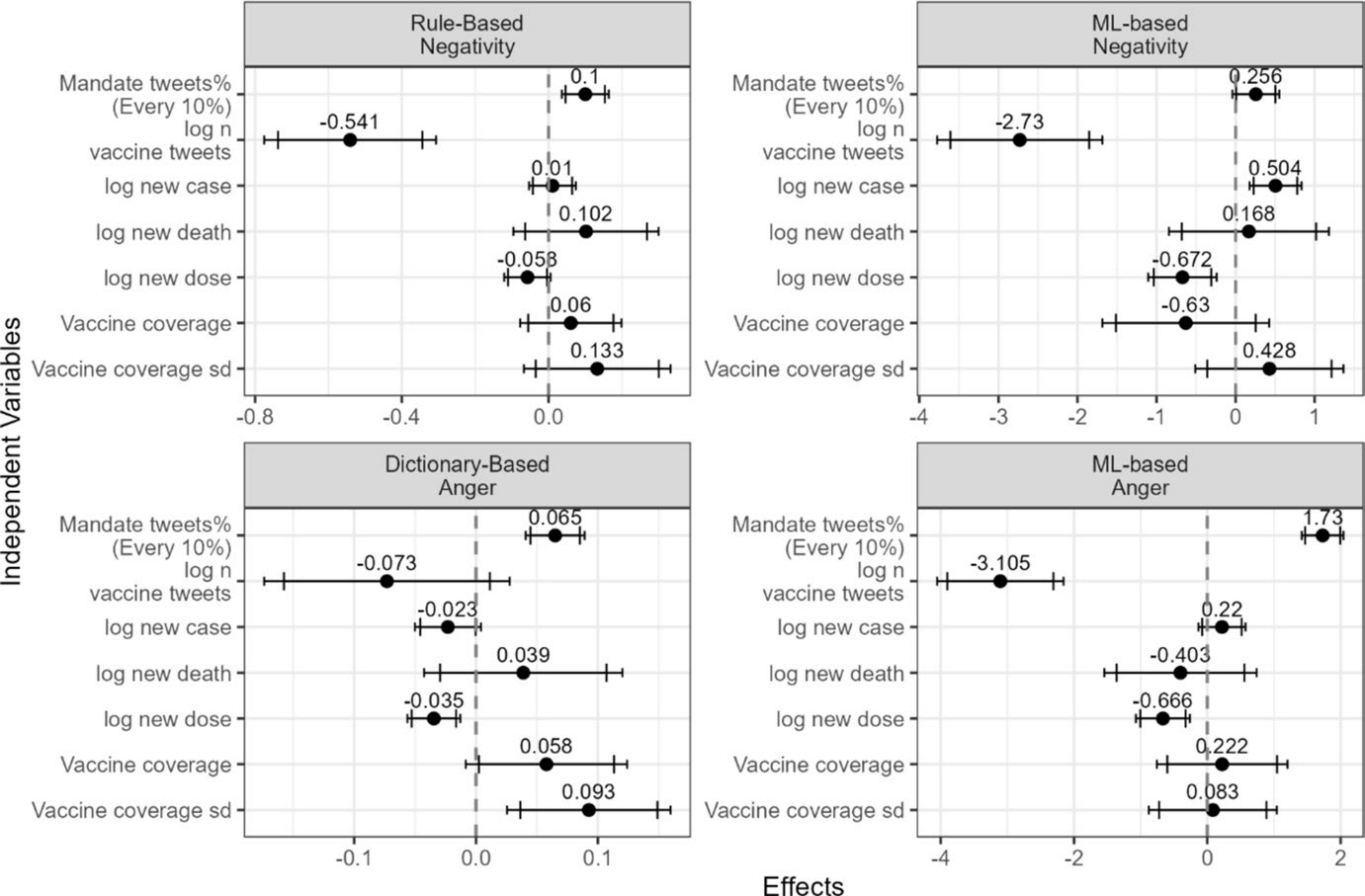
Regression results for vaccine-related tweets. The dependent variables are (1) average VADER negative sentiment score (top left), (2) percentage of negative tweets classified by TweetNLP (top right), (3) average percentage of anger words from the NRC Lexicon (bottom left), and (4) percentage of angry tweets classified by TweetNLP (bottom right). All model control for state and date fixed effects. Driscoll and Kraay’s version of panel Newey–West standard errors were used. The wider and narrower error bars present 90 and 95% confidence intervals, respectively

Consistent with the first hypothesis, a higher percentage of vaccine-related tweets contained freedom-related words when vaccine mandates were more salient (Fig. 3 and Table S2). This suggests that Twitter users were more likely to discuss freedom when vaccine mandates were a prominent topic. Figure 4 and Table S3 show evidence supporting the second hypothesis related to vaccine tweets. The mean negative sentiment score, as measured by the rule-based method, increased significantly in vaccine-related tweets within a state as the salience of vaccine mandates grew (Fig. 4, top left). The percentage of negative tweets, as classified by the machine-learning-based method, showed a marginally significant increase (Fig. 4, top right). With respect to anger, both measures of anger in the tweets increased significantly with greater salience of vaccine mandates (Fig. 4 bottom).

**Fig. 5:**
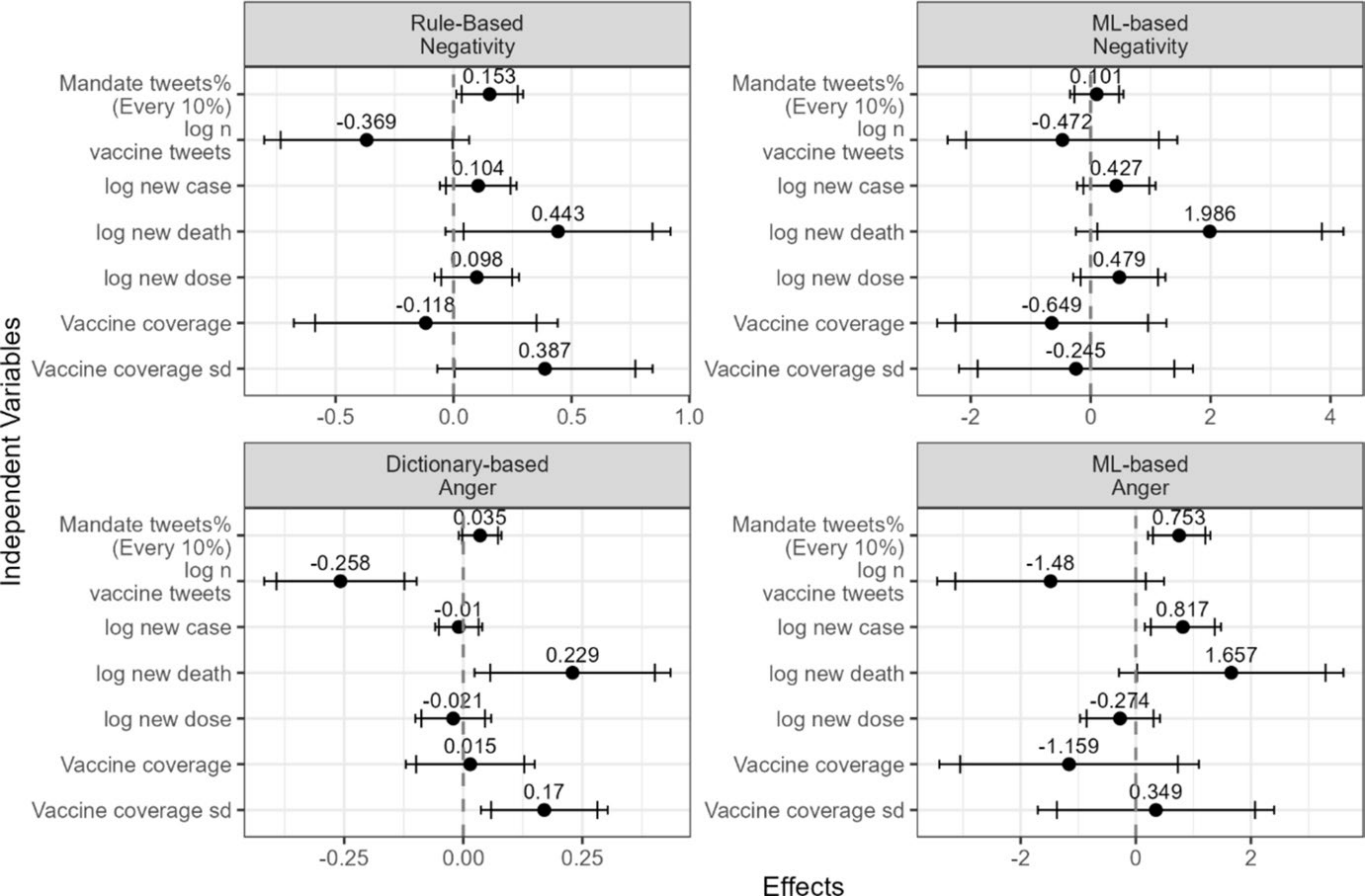
Regression results for tweets related to public health officials. The dependent variables are (1) average VADER negative sentiment score (top left), (2) percentage of negative tweets classified by TweetNLP (top right), (3) average percentage of anger words from the NRC Lexicon (bottom left), and (4) percentage of angry tweets classified by TweetNLP (bottom right). All model control for state and date fixed effects. Driscoll and Kraay’s version of panel Newey–West standard errors were used. The wider and narrower error bars present 90 and 95% confidence intervals, respectively

For tweets about public health officials, I found evidence supporting the third hypothesis (Fig. 5 and Table S5). Both negative sentiment measures and both anger measures increased with greater salience of vaccine mandates. The increase in the rule-based negative sentiment measure and the machine-learning-based anger measure was significant, while the other two measures were not. However, at least one measure of both negative sentiment and anger supports the third hypothesis.

The analysis revealed several other noteworthy relationships. A higher volume of vaccine-related tweets within a state was associated with less negativity in vaccine-related tweets and less anger in tweets about public health officials. Conversely, a higher seven-day moving average of new COVID-19 deaths per person was linked to increased anger in tweets directed toward public health officials. Interestingly, a greater number of new COVID-19 vaccine doses administered per person in the past seven days was associated with a decrease in freedom-related words and anger in vaccine-related tweets. Finally, the greater disparity in vaccination progress across counties within a state coincided with an increased likelihood of freedom and anger-related words in vaccine tweets and more anger in tweets about public health officials.

## Discussion

This study investigated the relationship between discussions surrounding vaccine mandates and psychological reactance on Twitter in the U.S. during the pandemic. By leveraging BERT, a large language model, tweets related to vaccine requirements were identified. Consistent with psychological reactance theory, tweets explicitly mentioning mandates exhibited a higher prevalence of freedom-related language and displayed a more negative and angry tone on average compared to non-mandate tweets. Furthermore, at the state level, an increased percentage of mandate-related tweets co-occurred with a rise in the likelihood of tweets about vaccines expressing freedom, as well as the levels of negativity and anger in tweets about vaccines and public health officials.

The results also demonstrate a nuanced relationship between negative sentiment, anger, and various COVID-19 metrics. First, vaccine-related tweets not concerning mandates did not contain expressions of negativity or anger toward vaccines or public health officials. Additionally, these non-mandate vaccine tweets were not associated with discussions of freedom. These findings suggest that the salience of vaccine mandates, rather than the salience of COVID-19 vaccines in general, is linked to negative sentiment and anger expressed in tweets about vaccines and public health officials.

Second, the disparity in COVID-19 vaccination rates across counties within a state emerged as another significant factor influencing the prevalence of freedom-related language and anger in discussions about vaccines and public health officials. This discord could potentially evoke anger from both those opposed to and in favor of vaccination. Moreover, research on psychological reactance posits that not only enforced mandates but also social pressure can trigger reactance [31]. Thus, the consequence can also be attributed to psychological reactance from social pressure.

The analysis revealed a positive association between the seven-day moving average of new COVID-19 deaths and anger expressed in tweets directed toward public health officials. This suggests that some Twitter users might have directed their anger toward those perceived as not following public health recommendations or, alternatively, expressed frustration with the perceived failure of public health officials to control the outbreak. However, no significant relationship was observed between COVID-19 deaths and negativity in vaccine-related tweets.

This study revealed the impact of discussions surrounding vaccine requirements, a proxy for the salience of these requirements to users, rather than the direct effects of implemented policies. There are three key reasons for this approach. First, the fragmented nature of vaccine requirements across government levels, private entities, and activities makes it difficult to quantify the affected population. For example, the state-wide mandates only impacted 2–13% of the population in those states. Nonetheless, as the following section demonstrates, Twitter discussions concerning vaccine mandates mirrored significant events in the U.S., such as the introduction of early state-level mandates and federal proposals. Second, the perception of coercion can arise even with proposed mandates, as public discourse often centers on potential policies regardless of formal implementation. Finally, research suggests that psychological reactance can be provoked when individuals perceive coercion of others, even if they themselves are not directly coerced [10, 32]. Therefore, focusing on discussions of vaccine requirements captures the effects when these requirements become salient to Twitter users, potentially offering a more comprehensive understanding than solely examining policy implementation.

The study has several limitations. First, Twitter users are not representative of the general U.S. population. Consequently, the findings cannot be generalized to suggest that the average American perceives vaccine mandates as coercive or experiences reactance. Rather, they illuminate the vocal sentiments of the Twitter community, which may not reflect broader societal attitudes. While acknowledging that Twitter users may not be representative of the entire U.S. population, social media analysis offers a valuable tool to examine reactions to significant events in a natural setting, free from researcher intervention [33]. The literature has also found that aggregated sentiments on Twitter reflect public sentiment at a community level [34–36].

Next, the design cannot rule out the possibility of reverse causality, in which a Twitter user wanted to tweet negatively and angrily about vaccines and public health officials and, in turn, mentioned vaccine mandates. It is possible that negative sentiment and anger toward vaccines and public health officials might have preceded a user’s decision to tweet about vaccine mandates. Nevertheless, this alternative explanation does not negate the observed association between opposition to vaccine mandates and these negative emotions.

Finally, the results from the rule-based and machine learning measures were not always consistent in this study, which can be attributed to several factors. First, machine learning models, especially natural language models pre-trained on large datasets, are generally better at capturing relationships between words and sentences than rule-based methods when they are properly validated and show strong performance in evaluations [37]. However, a key difference lies in the type of output: TweetNLP provides discrete labels, while VADER and the NRC Lexicon produce continuous measures. As a result, discrete labels may lose some of the variation present in the data (e.g., different levels of anger in tweets). Although TweetNLP outputs predicted probabilities, the model is still trained to predict discrete labels. In the future, I anticipate more machine learning models will be developed to handle continuous levels of sentiment and emotions, rather than discrete categories, to better capture these nuances.

## Conclusions

In the presented study, I found that the sentiment in tweets about vaccines and public health officials was associated with discussions of vaccine mandates. This suggests that it may not be the vaccines themselves or vaccination policies that do not involve mandates, such as financial incentives, but rather mandatory vaccination that induces resistance to vaccination and hostility toward public health officials. Despite the urgency of the COVID-19 pandemic, the potential effectiveness of mandatory vaccination must be weighed against potential drawbacks. While empirical evidence suggests increased vaccination rates following mandates [38, 39], such policies may also engender public resistance that could undermine societal resilience in future crises [5, 6, 40, 41]. Given that policymakers have access to a range of tools with varying levels of mandate [42], it is important to consider the long-term societal impacts of these policies. In a democratic society, public response to policies is particularly important, as citizen attitudes can significantly influence policy formulation. As we prepare for future public health challenges, understanding the broader implications of interventions beyond their immediate efficacy is crucial.

## Supplementary Information

Below is the link to the electronic supplementary material.

Supplementary file1 (PDF 152 KB)

## Data Availability

The data is available at 10.7910/DVN/JVGZGK. Tweet IDs, variables from text analysis, and state-level aggregate data are included. Data retrieved directly from the Twitter API, except for tweet IDs, cannot be shared in compliance with Twitter API policy.
