# Supplementary material for: Psychological reactance to vaccine mandates on Twitter: a study of sentiments in the United States
Source: J Public Health Policy. 2025 Jan 19;46(2):269–83. doi: 10.1057/s41271-025-00554-0 (PMC12119348; doi:10.1057/s41271-025-00554-0)
Supplement: Supplementary file 1 — Supplementary file1 (PDF 152 KB) [file 41271_2025_554_MOESM1_ESM.pdf]

Supporting Information for

Psychological Reactance to Vaccine Mandates on Twitter: A Study of Sentiments towards Vaccines and Public Health Officials on Twitter in the United States.

Appendix A. Additional Details for Training Machine Learning Models 3

Appendix B. Additional Comparisons between Mandate-Related and Non-Mandate-Related Tweets 4

Appendix C. Regression Table for State-Date Panel Data Analysis 6

Appendix A. Additional Details for Training Machine Learning Models

Four supervised learning models were employed for tweet classification of vaccine mandate discussions: Naive Bayes classifier, logistic regression with elastic net regularization, support vector machine (SVM), and BERTweet, a pre-trained BERT model for tweets. To train these models, a random sample of 3,324 tweets was manually labeled. Tweets discussing vaccination policies requiring inoculation to avoid restrictions (e.g., government- or private entity-imposed mandates for work or activities) were classified as mandate-related. The labeled data was randomly split in a 7:1.5:1.5 ratio for training, evaluation, and testing, respectively. This split accommodates the requirement of an independent evaluation set during BERTweet training. The remaining models (Naive Bayes, logistic regression with elastic net, and SVM) were trained on the combined training and evaluation sets.

All models were evaluated with the independent test set. The model evaluation shows that all the models have good overall accuracy. The accuracy of BERTweet, naive Bayes, logistic regression with the elastic net penalty, and SVM with a linear kernel are 93.15%, 84.41%, 87.45%, and 90.68%, respectively. However, as it is imbalanced data in which most tweets do not relate to mandates, a good model should also be able to capture as many mandate-related tweets as possible (i.e., have as few false negatives as possible). The BERTweet model's F1 score outperforms other models' F1 scores, a metric that considers both false positive rates and false negative rates (see Appendix A for the confusion matrix). Therefore, the BERTweet model was chosen to classify the remaining tweets.

		Predicted Label	
		Nonmandate related	Mandate related
True Label	Nonmandate related	69.2%\71.29%\72.24%\71.48%	3.99%\1.9%\0.95%\1.71%
	Mandate related	2.85%\13.69%\11.60%\7.60%	23.95%\13.12%\15.21%\19.20%

Table S1: The percentage in the test set (BERTweet\Naive Bayes\Logistic regression with the elastic net penalty\Support Vector Machine with a linear kernel

Appendix B. Additional Comparisons between Mandate-Related and Non-Mandate-Related Tweets

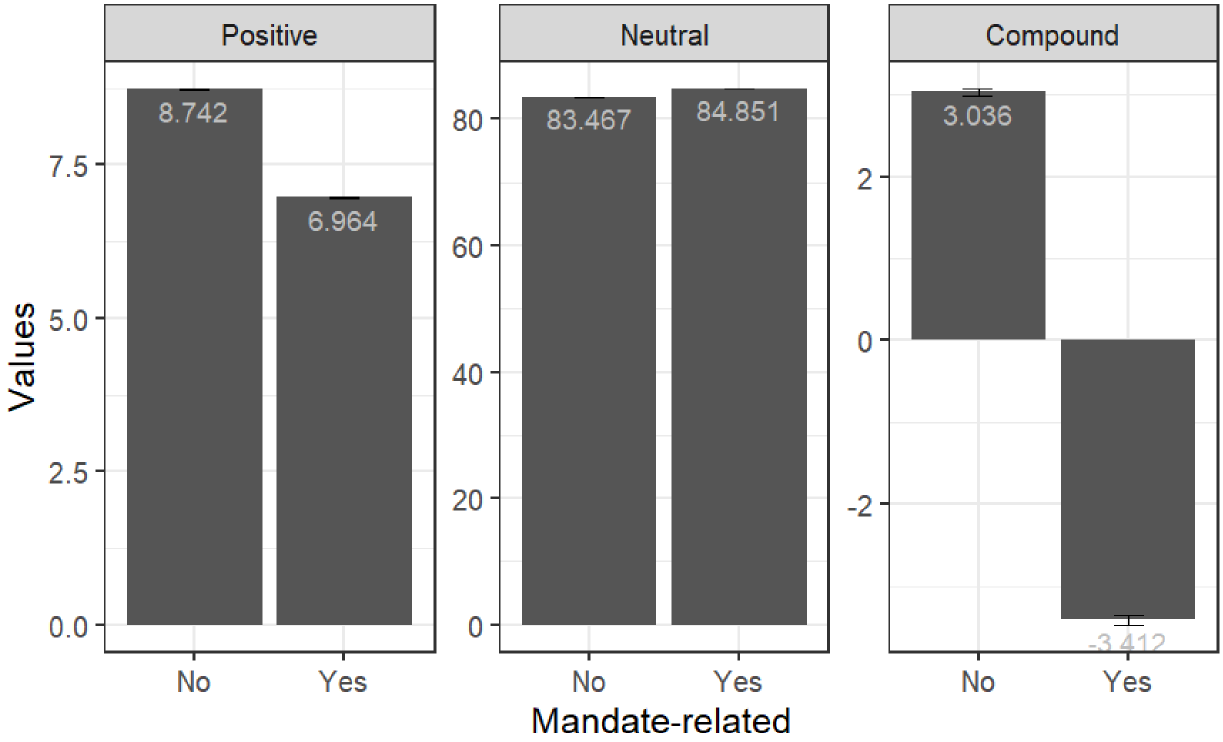

Figure S1: The comparison between mandate-related and other vaccine-related tweets. (Left): The mean of the VADER positive scores (from 0 to 100). (Middle): The mean of the VADER neutral scores (from 0 to 100). (Right): The mean of the VADER compound scores (from -100 to 100). 95% confidence interval for the error bars.

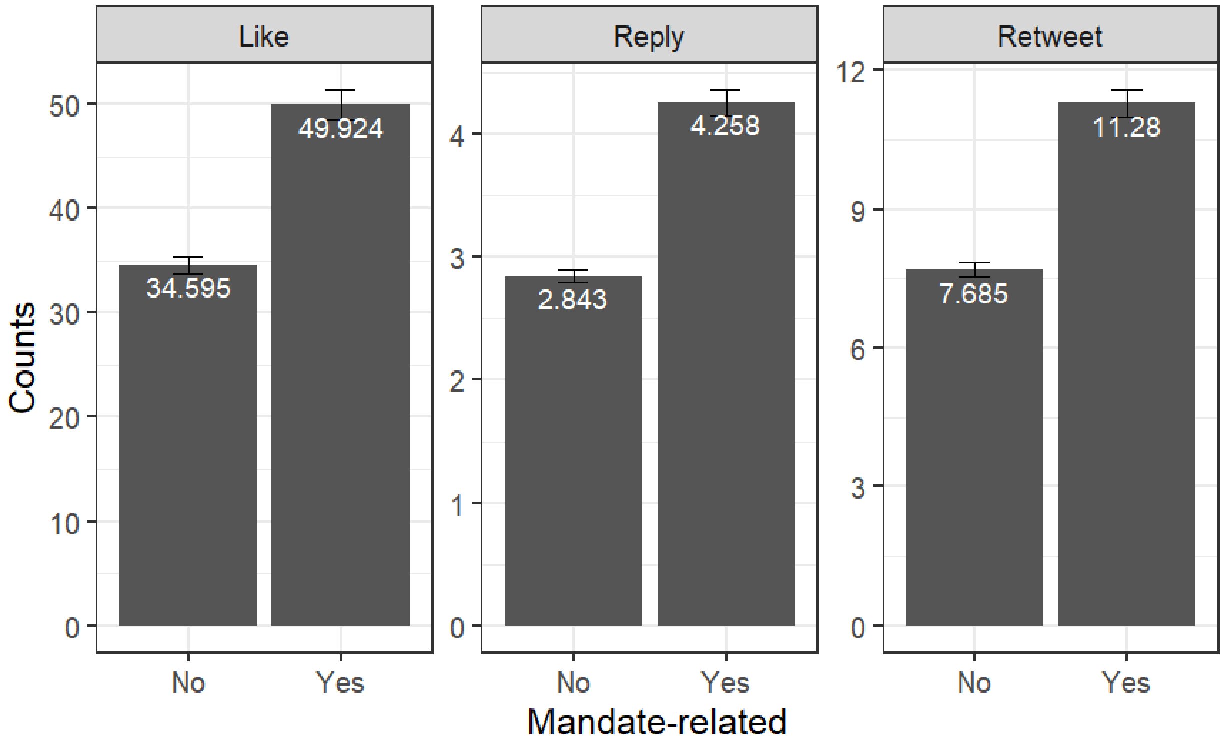

Figure S2: The comparison between mandate-related and other vaccine-related tweets. (Left): The average like counts. (Middle): The average reply counts. (Right): The average retweet counts. (95% confidence interval for the error bars.)

Appendix C. Regression Table for State-Date Panel Data Analysis

	Dependent variable:<br>% tweets mentioning freedom
Mandate tweets%<br>(Every 10%)	0.425***<br>(0.041)
log(100k vaccine tweets/person)	-0.243<br>(0.15)
log(new cases 7 day ma/person)	0.03<br>(0.052)
log(new deaths 7 day ma/person)	-0.131<br>(0.135)
log(new doses 7 day ma/person)	-0.159**<br>(0.052)
Fully vaccinated pop %<br>(every 10%)	-0.168<br>(0.132)
Weighted SD of fully<br>vaccinated pop % between counties	0.389*<br>(0.158)
State fixed effect	Yes
Date fixed effect	Yes
Observations	12,393
R^2	0.670

Note: The panel Newey-West standard errors are presented in brackets.
*+p<0.1;*p<0.05; **p<0.01; ***p<0.001*

Table S2. Regression results for the percentage of vaccine-related tweets containing freedom-related words. The dependent variable is the percentage of vaccine-related tweets that include a freedom-related word. The model controls for state and date fixed effects.

term	VADER negativity	TweetNLP negativity	NRCLex anger	TweetNLP anger
Mandate tweets% (Every 10%)	0.1**<br>(0.033)	0.256+<br>(0.151)	0.065***<br>(0.012)	1.73***<br>(0.16)
log(100k vaccine tweets/person)	-0.541***<br>(0.12)	-2.73***<br>(0.534)	-0.073<br>(0.051)	-3.105***<br>(0.484)
log(new cases 7 day ma/person)	0.01<br>(0.033)	0.504**<br>(0.168)	-0.023+<br>(0.014)	0.22<br>(0.18)
log(new deaths 7 day ma/person)	0.102<br>(0.101)	0.168<br>(0.516)	0.039<br>(0.042)	-0.403<br>(0.582)
log(new doses 7 day ma/person)	-0.058+<br>(0.032)	-0.672**<br>(0.221)	-0.035**<br>(0.011)	-0.666**<br>(0.207)
Fully vaccinated pop % (every 10%)	0.06<br>(0.071)	-0.63<br>(0.538)	0.058+<br>(0.034)	0.222<br>(0.5)
Weighted SD of fully vaccinated pop % between counties	0.133<br>(0.102)	0.428<br>(0.478)	0.093**<br>(0.034)	0.083<br>(0.489)
State fixed effect	Yes	Yes	Yes	Yes
Date fixed effect	Yes	Yes	Yes	Yes
Observations	12,393	12,393	12,393	12,393
R^2	0.981	0.981	0.965	0.980

Note: The panel Newey-West standard errors are presented in brackets.
+p<0.1;∗p<0.05; ∗∗p<0.01; ∗∗∗p<0.001

Table S3. Regression results for vaccine-related tweets. The model controls for state and date fixed effects.

	VADER negativity	TweetNLP negativity	NRCLex anger	TweetNLP anger
Mandate tweets% (Every 10%)	0.153* (0.072)	0.101 (0.227)	0.035 (0.023)	0.753** (0.276)
log(100k vaccine tweets/person)	-0.369+ (0.222)	-0.472 (0.978)	-0.258** (0.082)	-1.48 (1.005)
log(new cases 7 day ma/person)	0.104 (0.083)	0.427 (0.334)	-0.01 (0.025)	0.817* (0.338)
log(new deaths 7 day ma/person)	0.443+ (0.243)	1.986+ (1.139)	0.229* (0.105)	1.657+ (0.994)
log(new doses 7 day ma/person)	0.098 (0.091)	0.479 (0.393)	-0.021 (0.041)	-0.274 (0.354)
Fully vaccinated pop % (every 10%)	-0.118 (0.285)	-0.649 (0.976)	0.015 (0.069)	-1.159 (1.149)
Weighted SD of fully vaccinated pop % between counties	0.387+ (0.233)	-0.245 (0.997)	0.17* (0.068)	0.349 (1.046)
State fixed effect	Yes	Yes	Yes	Yes
Date fixed effect	Yes	Yes	Yes	Yes
Observations	12,367	12,367	12,367	12,367
R^2	0.894	0.897	0.779	0.898

Note: The panel Newey-West standard errors are presented in brackets.
+p<0.1;∗p<0.05; ∗∗p<0.01; ∗∗∗p<0.001

Table S4. Regression results for tweets related to public health officials. The model controls for state and date fixed effects.
